# Cost-effectiveness analysis of malaria rapid diagnostic tests: a systematic review

**DOI:** 10.1186/s40249-019-0615-8

**Published:** 2019-12-30

**Authors:** Xiao-Xiao Ling, Jia-Jie Jin, Guo-Ding Zhu, Wei-Ming Wang, Yuan-Yuan Cao, Meng-Meng Yang, Hua-Yun Zhou, Jun Cao, Jia-Yan Huang

**Affiliations:** 1 School of Public Health, Fudan University, Key Laboratory of Health Technology Assessment, National Health Commission, Shanghai, 200032 China; 2 National Health Commission Key Laboratory of Parasitic Disease Control and Prevention, Jiangsu Provincial Key Laboratory on Parasite and Vector Control Technology, Jiangsu Institute of Parasitic Diseases, Wuxi, 214064 China; 3 Center for Global Health, School of Public Health, Nanjing Medical University, Nanjing, 211166 China; 4 Public Health Research Centre, Jiangnan University, Wuxi, 214122 China

**Keywords:** Malaria, Rapid diagnostic test, Microscopy, Presumptive diagnosis, Cost-effectiveness analysis

## Abstract

**Background:**

Rapid diagnostic tests (RDT) can effectively manage malaria cases and reduce excess costs brought by misdiagnosis. However, few studies have evaluated the economic value of this technology. The purpose of this study is to systematically review the economic value of RDT in malaria diagnosis.

**Main text:**

A detailed search strategy was developed to identify published economic evaluations that provide evidence regarding the cost-effectiveness of malaria RDT. Electronic databases including MEDLINE, EMBASE, Biosis Previews, Web of Science and Cochrane Library were searched from Jan 2007 to July 2018. Two researchers screened studies independently based on pre-specified inclusion and exclusion criteria. The Consolidated Health Economic Evaluation Reporting Standards (CHEERS) checklist was applied to evaluate the quality of the studies. Then cost and effectiveness data were extracted and summarized in a narrative way.

Fifteen economic evaluations of RDT compared to other diagnostic methods were identified. The overall quality of studies varied greatly but most of them were scored to be of high or moderate quality. Ten of the fifteen studies reported that RDT was likely to be a cost-effective approach compared to its comparisons, but the results could be influenced by the alternatives, study perspectives, malaria prevalence, and the types of RDT.

**Conclusions:**

Based on available evidence, RDT had the potential to be more cost-effective than either microscopy or presumptive diagnosis. Further research is also required to draw a more robust conclusion.

## Background

For years, the quality-assured malaria diagnosis has been emphasized to effectively control malaria cases and reduce excess costs due to misdiagnosis [1–4]. With the development of malaria control interventions and the shift towards malaria elimination globally, many countries face the new challenge of increasing imported cases due to the growing human migration and travel to the malaria-endemic region [5–7]. Failing to identify and track malaria cases promptly may hinder the realization of disease elimination and impose a substantial financial burden given the higher treatment costs and public spending. This arouses a wide concern among policymakers regarding how available malaria diagnostic methods can achieve the goal of the disease control and elimination, and whether existing packages of feasible interventions can be sustainably affordable [8].

So far, a remarkable improvement in the malaria diagnosis has been observed. Several malaria diagnostic methods are available for policymakers to choose: presumptive diagnosis, blood smear microscopy, polymerase chain reaction (PCR), and rapid diagnostic test (RDT) [9]. The presumptive diagnosis of malaria is a conventional approach that diagnoses patients based on their symptoms and clinical signs and it is still widely adopted [10]. However, it has been acknowledged that the method may add to the difficulty in effectively and accurately diagnosing the disease, and lead to a high proportion of misdiagnosis and overuse of drugs. Blood smear microscopy has advantages in both accuracy and ability to quantify parasites if it could be used properly [11]. But it has high requirements for technicians’ skills and experience, which is difficult to guarantee especially in low transmission sites [12]. It also takes a longer time to operate, far from current expectations of an accurate and timely technique for routine malaria detection [13]. PCR is appealing for its high diagnostic accuracy. On the other side, it is most costly and has high requirements on devices, materials, and technicians, making it inappropriate for countries with limited resources [14–16].

Rapid diagnostic test (RDT) is a quick diagnostic approach to detect malaria among malaria-suspected patients and rule out malaria among individuals without malaria. It has been found that the test is highly sensitive and specific [17]. Meanwhile, RDT is easy to perform, and the results can be read in 15–30 min. These make it suitable for community-level health facilitates in rural areas and other endemic situations where equipment and professional microscopists are not accessible.

Given the rapid development of malaria diagnosis, enhanced case identification is operationally feasible but now the question of concern for countries embarking on malaria control and elimination is how to allocate limited resources to strengthen their current surveillance system, maintain their success and avoid the risk of re-introduction of malaria particularly when the cost is largely unknown [18]. Economic evaluations can provide evidence for policymakers to identify the diagnostic test that is cost-effective and can be sustainably applied. Few studies have been carried out to evaluate the economic value of RDT, although economic evidence is necessary, and no systematic review has been performed. This study focuses on both the costs and effects of RDT and systematically evaluates whether using RDT can be cost-effective compared with other malaria diagnostic methods based on available evidence.

## Methods

### Selection criteria

To assess the cost-effectiveness (cost-utility or cost-benefit), we only considered full economic evaluations that compared RDT with other common malaria diagnostic tools. A full economic evaluation should consist of two parts, i.e. costs and effects, and provide resource use, estimates of inputs and consequences for intervention. Studies were excluded if they did not use microscopy or PCR as the reference for malaria diagnosis.

### Search strategy

The search was performed initially in March 2017 and updated in July 2018 in the following databases: Cochrane Library, MEDLINE, EMBASE, Web of Science and Biosis Previews. We determined our search strategy with reference to previous relevant studies and systematic reviews. It was segmented into three components: malaria, malaria diagnosis techniques, and economic evaluations. To maintain the search comprehensiveness, the search was restricted to articles with the following terms in their titles, abstracts and keywords: “malaria”, and “RDT”, or “rapid diagnosis test”, and “cost-effectiveness”, “cost-effectiveness analysis”, “cost-benefit analysis”, “cost-utility analysis”, “economic evaluations”, “cost(s)”, or “economy”. We did not set limitations on population and languages.

### Selection of studies

Two reviewers independently screened the titles, abstracts, and keywords of all searched studies and excluded irrelevant studies based on selection criteria. Then duplicates were removed. Two reviewers independently read the full-text version of each study and decided whether they should be included. Disagreements on inclusion were resolved by discussion or inviting another reviewer to judge according to the same information. Studies were named by the surname of their first author and the year of publication.

### Data extraction

Two reviewers independently extracted data based on a well-designed data extraction table, summarized important information and made a descriptive analysis to draw a conclusion about the cost-effectiveness of RDT.

The following data were extracted:

1. General information: first author, study year, country, the prevalence of malaria, source of funding, participants of the study, intervention and its comparisons, commercial name of RDT, the type of RDT.
2. Methodological information: types of study (cost-effectiveness analysis, cost-utility analysis, and cost-benefit analysis), study design, study perspective, time horizon, outcome measures, discount rate, currency, price year and willingness to pay threshold.
3. Results and conclusions: incremental costs, incremental effectiveness, incremental cost-effectiveness ratio (ICER) reported, and sensitivity analysis.

### Quality assessment

Strict quality assessment can reflect the methodological quality of health economics research and control risk of bias. The Consolidated Health Economic Evaluation Reporting Standards (CHEERS, http://www.equator-network.org/wp-content/uploads/2013/04/Revised-CHEERS-Checklist-Oct13.pdf), a comprehensive quality assessment tool recommended by the International Society for Pharmacoeconomics and Outcomes Research, were used to examine the quality of studies. The CHEERS checklist assesses the reporting quality based on items from the following aspects: “title and abstract”, “introduction”, “methods”, “discussion” and “other”. Each item would be appraised critically in accordance with the requirements of CHEERS statements. The evaluation results were shown as ‘Yes’, ‘No’ and ‘Not clear’ marked as 1, 0 and 0 respectively, then the scores of the 24 items were summed up to calculate the final score of each study. Scores would be divided into three levels to identify the quality of each article: high (studies that met over 75% items or scored between 19 and 24), moderate (studies that met 50–75% items or scored between 13 and 18) and low (studies that met less than 50% items or scored 12 or lower).

### Analysis methods

As meta-analysis or other quantitative synthesis methods are not recommended to combine the cost-effectiveness from different economic evaluations [19], we summarized results of each study in a narrative way and presented incremental costs, incremental effectiveness, and ICERs in structured tables if such information was available.

The narrative and tabular summary were structured based on the age of the population and the perspective of economic evaluations since participants’ age might influence the economic results of the intervention [20–22], and the perspectives could determine the costs and effects that were included in the evaluations. We also recognized that there might be potential impact brought by the type of RDTs and funding sources on the economic values of diagnostic tests and took it into account in our analysis.

To facilitate the comparison across different studies, original costs reported were converted to a common currency and price year, 2019 United States dollars (USD), given the latest version of a web-based cost converter. This tool adjusts cost estimates based on purchasing power parity (PPP) and gross domestic product (GDP) deflator index and has been recommended by the guidelines of systematic review [19, 23]. ICERs were then recalculated by dividing the converted costs by the original effects. In order to graphically compare the economic value of different malaria diagnostic methods, we adopted the cost-effectiveness plane to reflect the differences in ICER, the only appropriate measure that can capture the true economic value [24], for studies that provided relevant data and took the same outcomes of effectiveness. Studies would be categorized according to the perspectives of economic evaluations. We recognized that taking narrower perspectives (e.g., a provider perspective) could impede the comparability of the results throughout healthcare systems and ignore the implication of opportunity costs brought by the introduction of new technologies [25, 26]. Thus we selected results from studies taken the societal perspective and the same outcome measures and plotted them on the same cost-effectiveness plane.

## Results

**Fig. 1:**
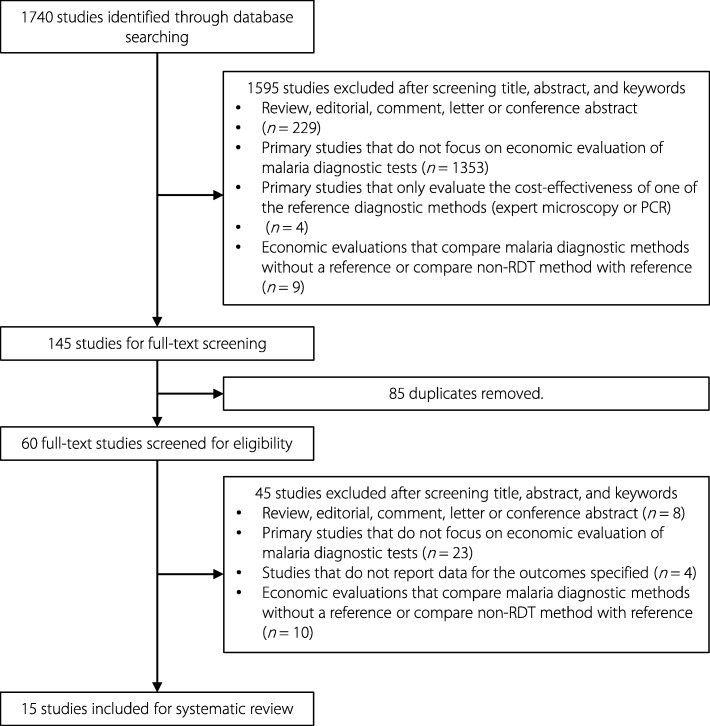
Study flow diagram. Flowchart showing inclusion and exclusion process of study identification. RDT: Rapid diagnostic test

One thousand seven hundred forty studies were identified from electronic databases. After screening the titles, abstracts, and keywords, 1595 studies were removed based on inclusion and exclusion criteria, 85 studies were removed due to the duplicates and 60 full-text potentially eligible articles were retrieved for the consideration. Finally, 15 studies were included in the analysis [27–41]. The flow diagram of our study selection is shown in Fig. 1.

### General characteristics of studies

We included fifteen studies that compared the economic value of RDT with other malaria diagnostic methods. Fourteen studies were full health economic evaluations that made a comparison in terms of costs and effectiveness between RDT and its comparators. All of them were cost-effectiveness analyses, nine of which used decision tree models. Besides, one study, although did not say that it was a cost-effectiveness analysis, assessed both the costs and the specificity of RDT, thus we also considered it as full economic evaluations and included it [28] (Table 1).

**Table 1:** General characteristics of studies included

Study ID	Study Year	Country	Prevalence of malaria	Study Type	Design	Participants	Intervention	Commercial name of RDT	Types of RDT	Quality	Quality class
Batwala 2011 [27]	2010/03–2011/02	Uganda	High/Low	CEA	Decision tree	22 052 fever outpatients	microscopy			13	Moderate
							RDT	Paracheck	Single		
Gitonga 2012 [28]	2008/09–2010/03	Kenya	Stable & seasonal transmission	Cost analysis	Cross-sectional study	49 891 students	microscopy			7	Low
							RDT	OptiMal - IT	Single		
								Paracheck - Pf device	Single		
								Paracheck - Pf dipstick	Single		
								CareStart - Pf/Pv combo	Combo		
Hansen 2015 [29]	2009/09–2010/09	Afghanistan	Moderate/Low	CEA	Decision tree	5749 suspected malaria patients	RDT	CareStart Malaria RDT Pf/Pan	Combo	22	High
Hansen 2017a [30]	2011/01–2011/12	Uganda	Not clear	CEA	Decision tree	13 319 customers suspected malaria and visiting drug shops	RDT	First Response	Single	15	Moderate
Hansen 2017b [31]	2011/01–2011/12	Uganda	Moderate to high/Low	CEA	Decision tree	Children under five visiting CHWs	RDT	First Response	Single	12	Low
Lemma 2011 [32]	2007	Ethiopia	Not clear	CEA	Cross-sectional study	2422 malaria suspected patients	RDT	Paracheck - pf	Single	11	Low
								Parascreen - pan/pf	Combo		
Lubell 2007 [33]	2005	Tanzania	High/Low	CEA	RCT	2416 patients requested for a parasitological test	RDT	Paracheck - pf	Single	10	Low
Ly 2010 [34]	2008/10–2009/01	Senegal	High/Moderate	CEA	Cross-sectional study	189 suspected malaria patients	RDT	Paracheck - pf	Single	12	Low
Matangila 2014 [35]	2012/07–2012/08	Congo	Not clear	CEA	Cross-sectional study	332 pregnant women	microscopy			18	Moderate
							RDT	SD Bioline Malaria Ag Pf	Single		
Oliveira 2010 [36]	2006	Brazil	Not clear	CEA	Decision tree	33 491 individuals with fever	microscopy			15	Moderate
							RDT	OptiMal	Combo		
Oliveira 2012 [37]	2010	Brazilian Extra-Amazon	Low	CEA	Decision tree	2702 suspected patients who took the diagnostic tests in Extra-Amazon region in 2010	RDT (5 brands)	SD Bioline FK60 (PF/Pan)	Combo	11	Low
								CareStart (Pan)			
								First Response			
								ParascreenTM (Pf/Pan)			
								ICT BinaxNOW Malaria			
Osei-Kwakye 2013 [38]	2009/01–2010/02	Ghana	High	CEA	Cross-sectional study	936 children under five years with fever at the outpatient department	microscopy			18	Moderate
							RDT	Parascreen	Combo		
Shillcutt 2008 [39]	NR	Sub-Saharan endemic countries	All levels	CEA	Decision tree	A hypothetical cohort of outpatients with fever in rural area of sub-Saharan Africa	microscopy			11	Low
							RDT	a hypothetical HRP2-based RDT for *P. falciparum*	Single		
Tawiah 2016 [40]	NR	Ghana	High	CEA	Decision tree	100 children under 24 months per health center in total 32 health centers	RDT	CareStart		23	High
								First Response	Single		
Uzochukwu 2009 [41]	2005–2007	Nigeria	High	CEA	Decision tree	638 patients with fever, diagnosed as malaria	microscopy			11	Low
							RDT	ICT Malaria Combo Cassette Test	Combo		

*NR* Not report, *SNMCP* The Senegalese National Malaria Control Programme, *CEA* Cost-effectiveness analysis, *RCT* Randomised controlled trial, *RDT* Rapid diagnostic test, *IT* Individual test, *Pf* *Plasmodium falciparum*, *Pv* *Plasmodium vivax*, *CHW* Community health workers

Quality: the reporting quality of each study identified based on CHEERS checklist with a maximum score of 24. Quality class: quality rating was divided into three categories based on scores: high (19–24), moderate (13–18) and low (0–12)

Most of the studies were conducted in Africa, except three: one in Afghanistan [29], and two in Brazil [36, 37]. The Africa-based studies were all performed in Sub-Saharan Africa (Ethiopia [32], Congo [35], Ghana [38, 40], Kenya [28], Nigeria [41], Senegal [34], Tanzania [33], Uganda [27, 30, 31]). One study targeted at all endemic countries in Sub-Saharan Africa using a simulated cohort with fever in the rural areas [39].

Eleven studies focused on suspected malaria and fever patients. Among the other four studies, two targeted at children [38, 40], one focused on the application of RDT in school students [28], and one assessed the effectiveness of RDT among healthy pregnant women [35].

### Quality assessment

**Fig. 2:**
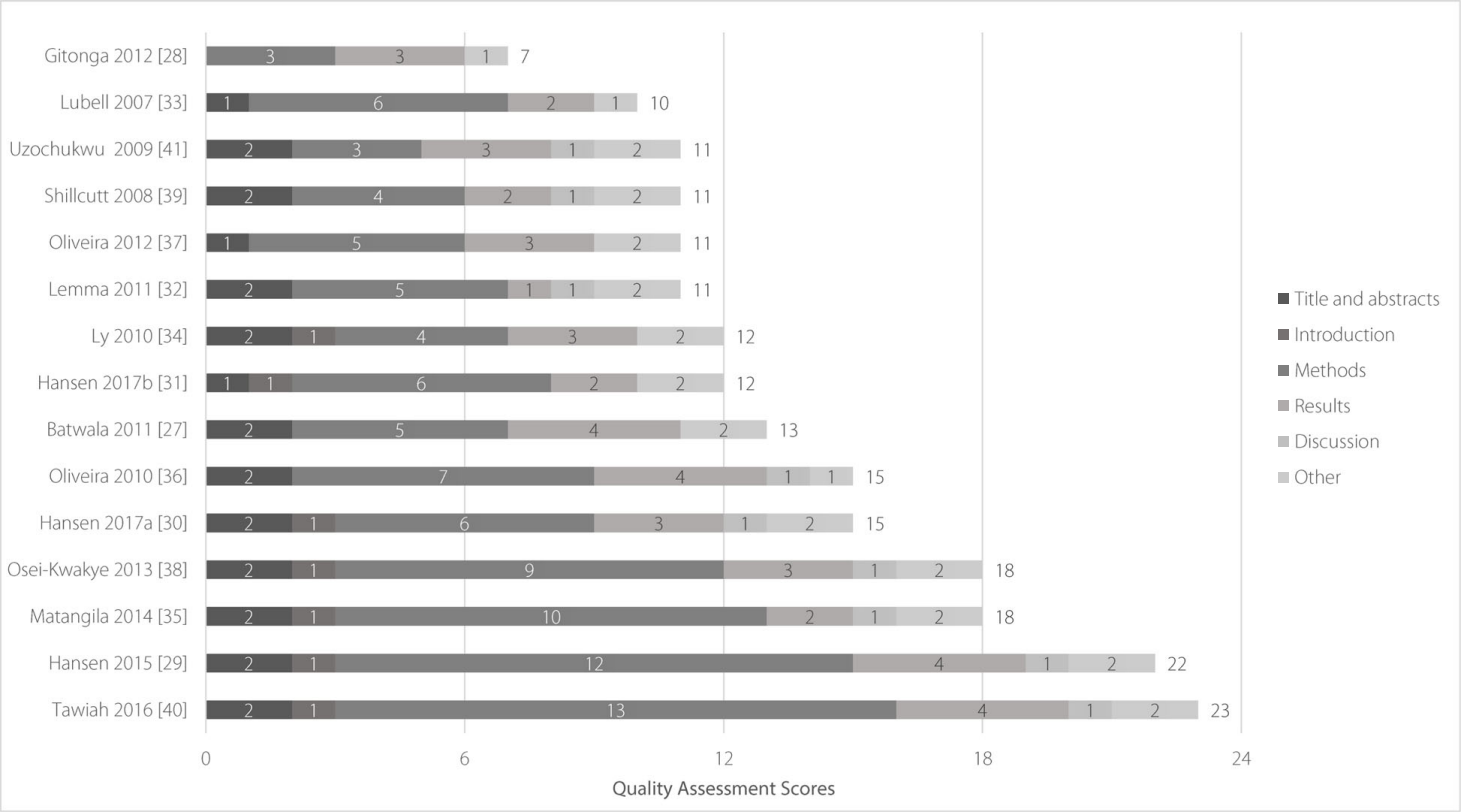
Quality assessment results of studies included The quality of each included study was assessed based on the 24-item CHEERS checklist with a maximum score of 24 if the study could meet quality criteria for all the items.

According to the CHEERS checklist, huge gaps existed in the quality of evidence reported. Scores ranged from 7 to 23. Two studies provided a high quality of evidence with the highest score of 23 [29, 40], five had evidence of moderate quality [27, 30, 35, 36, 38], and eight had low quality with the lowest score of 7 [28, 31–34, 37, 39, 41]. The overall quality of all studies included could be seen in Fig. 2 and Additional file 1.

### The cost-effectiveness of RDT

The economic value of RDT was assessed in the fifteen economic evaluations and summarized in Table 2. Three malaria diagnostic techniques were reported and compared in all papers: RDT, microscopy, and presumptive diagnosis, and the majority took microscopy and/or presumptive diagnosis method as the comparison for RDT.

**Table 2:** Summaries of economic results of included studies

Study ID	Study perspective	Time horizon	Effectiveness measures	Incremental costs	Incremental effectiveness	ICERs	Sensitivity analysis	Willingness-to-pay threshold	Price year	Discount rate
Batwala 2011 [27]	Societal	12 months	The number and proportion of patients correctly diagnosed and treated	RDT vs presumptive: USD 1.17	RDT vs presumptive: 0.234	Incremental cost per patient correctly diagnosed and treated of replacing presumptive diagnosis by RDT was USD5.0; and by microscopy was USD 9.61. In high transmission setting, the ICER was USD 4.38 for RDT and was USD 12.98 for microscopy. In low transmission setting, the ICER was USD 5.85 for RDT and USD 7.63 for microscopy.	Reduction in the cost of AL and RDT, and increase in malaria prevalence were associated with improvement in the cost-effectiveness of RDT.	USD 2.8	USD 2011	3%
				RDT vs microscopy: USD - 0.31	RDT vs microscopy: 0.08					
Gitonga 2012 [28]	NR	NR	The percentage of districts in a given prevalence that were correctly classified	NR	NR	The incremental analysis was not performed	NR	NR	USD 2008–2010	3%
Hansen 2015 [29]	Societal	12 months	Appropriate treatment of suspected malaria	RDT vs presumptive (low transmission): USD 2.4	RDT vs presumptive (low transmission): 53.4%	Incremental cost per appropriately treated patient of replacing presumptive diagnosis by RDT was USD 4.5 from a societal perspective.	Probabilistic sensitivity analysis: RDT vs presumptive - Improved effects compared but uncertainty in the incremental costs. RDT vs microscopy - In moderate transmission setting, improved effects in RDT but uncertainty in the incremental costs; In low transmission setting, lower costs in RDT but uncertainty in the effects. Scenario analysis: RDT remained cost-effective compared to microscopy if chloroquine was replaced by ACT or the price of ACT increased.	NR	USD 2009	3%
				RDT vs microscopy (moderate transmission): USD - 0.3 RDT vs microscopy (low transmission): USD - 7.1	RDT vs microscopy (moderate transmission): 7.4% RDT vs microscopy (low transmission): 4%	Incremental cost per appropriately treated patient of replacing microscopy by RDT from a societal perspective was USD - 4.1 (dominant) in moderate transmission setting and USD - 177.5 (dominant) in low transmission setting.				
	Health sector			RDT vs presumptive (low transmission): USD 1.3	RDT vs presumptive (low transmission): 53.4%	Incremental cost per appropriately treated patient of replacing presumptive diagnosis by RDT was USD 2.5 from a health sector perspective.				
				RDT vs microscopy (moderate transmission): USD - 0.0 RDT vs microscopy (low transmission): USD - 7.1	RDT vs microscopy (moderate transmission): 7.4% RDT vs microscopy (low transmission): 4%	Incremental cost per appropriately treated patient of replacing microscopy by RDT from a health sector perspective was USD - 0.0 (dominant) in moderate transmission setting and USD - 177.5 (dominant) in low transmission setting.				
Hansen 2017a [30]	Societal	12 months	Appropriate treatment of malaria with ACT or rectal artesunate	RDT vs presumptive: USD 1658	RDT vs presumptive: 433	Incremental cost per additional patient appropriately treated of malaria for RDT compared to presumptive diagnosis was USD 3.83 from a societal perspective	Univariate sensitivity analysis: ICER was sensitive to malaria prevalence levels, RDT price, the specificity of RDT, higher popularity of drug shops offering RDT, adherence to RDT results and ACT prices. Probabilistic sensitivity analysis: Improved effects of RDT. Increased costs of RDT from a health sector perspective and uncertainty in incremental costs from a societal perspective	NR	USD 2011	3%
	Health sector			RDT vs presumptive: USD 239		Incremental cost per ` patient appropriately treated of malaria was USD 0.55 from a health sector perspective				
Hansen 2017b [31]	Societal	12 months	Appropriate treatment of malaria with ACT	RDT vs presumptive (moderate to high transmission): USD 1755	RDT vs presumptive (moderate to high transmission): 485	In moderate-to-high transmission setting, incremental cost per additional appropriately treated child under five from a societal perspective was USD 3.6.	Univariate sensitivity analysis: ICER was sensitive to malaria prevalence levels, RDT price, adherence to RDT results and the number of children visiting community health workers. Probabilistic sensitivity analysis: Improved effects of RDT but also increased costs	NR	USD 2011	3%
				RDT vs presumptive (low transmission): USD 12283	RDT vs presumptive (low transmission): 822	In low transmission setting, incremental cost per additional appropriately treated child under five from a societal perspective was USD 14.9.				
	Health sector			RDT vs presumptive (moderate to high transmission): USD 1462	RDT vs presumptive (moderate to high transmission): 485	In moderate-to-high transmission setting, incremental cost per additional appropriately treated child under five from a health sector perspective was USD 3.0.				
				RDT vs presumptive (low transmission): USD 10924	RDT vs presumptive (low transmission): 822	In low transmission setting, incremental cost per additional appropriately treated child under five from a health sector perspective was USD 13.3.				
Lemma 2011 [32]	Provider	NR	The number of correctly treated cases	RDT (Parascreen) vs presumptive: USD - 1388.44	RDT (Parascreen) vs presumptive: 1690	Incremental cost on Parascreen-BS over presumptive was USD - 0.82	Result robust: presumptive diagnosis was always dominated.	NR	USD 2007	NR
				RDT (Paracheck) vs presumptive: USD - 2312.71	RDT (Paracheck) vs presumptive: 127	Incremental cost on Paracheck-BS over presumptive was USD - 18.21				
Lubell 2007 [33]	Provider	NR	The proportion of patients correctly treated	RDT vs microscopy (high transmission): USD 0.6	RDT vs microscopy (high transmission): 9.4%	In high transmission setting, incremental cost per additional patient correctly treated was USD 7.0	ICER was sensitive to malaria prevalence and the price of RDT. Result was robust to the cost of ACT.	NR	USD 2005	NR
				RDT vs microscopy (low transmission): USD 0.6	RDT vs microscopy (low transmission): 2.3%	In low transmission setting, incremental cost per additional patient correctly treated was USD 25.2				
Ly 2010 [34]	SNMCP	NR	The proportion of patients would have been correctly managed	Treatment of all RDT positive patients vs presumptive treatment of all the febrile patients based on their body temperature: $ 460.16 (€ 336.3) per 1000 episodes	Treatment of all RDT positive patients vs presumptive treatment of all the febrile patients based on their body temperature: 48.1%	Incremental cost per additional episode of illness correctly managed of treatment of all RDT positive patients compared to presumptive treatment of all the febrile patients based on their body temperature was $ 0.96 (€ 0.699)	The cost would increase around 50% with full adherence to the test results.	NR	EUR 2008–2009	NR
				Treatment of all RDT positive patients vs presumptive treatment of suspected patients based on health care provider’s feeling: $ 448.12 (€ 327.5) per 1000 episodes	Treatment of all RDT positive patients vs presumptive treatment of suspected patients based on health care provider’s feeling: 46.6%	Incremental cost per additional episode of illness correctly managed of treatment of all RDT positive patients compared to presumptive treatment of suspected patients based on health care provider’s feeling was $ 0.96 (€ 0.703)				
				Treatment of all RDT positive patients vs treatment of all febrile RDT positive patients: $ 484.10 (€ 353.8) per 1000 episodes	Treatment of all RDT positive patients vs treatment of all febrile RDT positive patients: 2.6%	Incremental cost per additional episode of illness correctly managed of treatment of all RDT positive patients compared to treatment of all febrile RDT positive patients was $ 18.62 (€ 13.608)				
				Treatment of all RDT positive patients vs treatment of all children under 6 and all RDT positive patients above 6: $ 119.73 (€ 87.5) per 1000 episodes	Treatment of all RDT positive patients vs treatment of all children under 6 and all RDT positive patients above 6: 40.2%	Incremental cost per additional episode of illness correctly managed of treatment of all RDT positive patients compared to treatment of all children under 6 and all RDT positive patients above 6 was $ 0.30 (€ 0.218)				
Matangila 2014 [35]	Provider	1 month	The number of cases correctly diagnosed	RDT vs microscopy: USD - 1.46	RDT vs microscopy: 2.3%	Incremental cost per additional case correctly diagnosed for RDT compared to microscopy was USD - 63.47.	Sensitivity analysis for the incremental analysis was not performed.	NR	USD 2012	NR
Oliveira 2010 [36]	Public health system	12 months	The number of adequate diagnosis of suspected malaria	RDT vs microscopy: USD -1.65	RDT vs microscopy: - 0.3%	Incremental cost per additional case correctly diagnosed for RDT compared to microscopy was USD 549.92	ICER was sensitive to the sensitivity and specificity of microscopy, the specificity of RDT, the cost of RDT, the cost of transportation to perform one rapid test and thick smear.	NR	USD 2006	5%
Oliveira 2012 [37]	Public health system	12 months	Adequately diagnosed cases of malaria	RDT (First Response malaria combo) vs exclusive-use microscopy: USD - 24.37	RDT (First Response malaria combo) vs exclusive-use microscopy: - 0.0685	Incremental cost per additional adequately diagnosed by First Response Malaria Combo compared to exclusive-use microscopy was USD 355.77	ICERs of ICT BinaxNOW and CareStart in relation to both exlusive- and shared-use microscopy were robust to the cost of RDT. The cost-effectiveness of SD Bioline was sensitive to the malaria prevalence when RDTs were compared with exclusive-use microscopy. The cost-effectiveness of shared-use microscopy was sensitive to the sensitivity to P.vivax of microscopy and RDT.	NR	USD 2010	NR
				RDT (Parascreen) vs exclusive-use microscopy: USD - 24.27	RDT (Parascreen) vs exclusive-use microscopy: - 0.1141	Incremental cost per additional adequately diagnosed by Parascreen compared to exclusive-use microscopy was USD 212.71				
				RDT (SD Bioline FK60) vs exclusive-use microscopy: USD - 24.26	RDT (SD Bioline FK60) vs exclusive-use microscopy: - 0.0767	Incremental cost per additional adequately diagnosed by SD Bioline FK60 compared to exclusive-use microscopy was USD 316.30				
				RDT (CareStart) vs exclusive-use microscopy: USD - 21.33	RDT (CareStart) vs exclusive-use microscopy: - 0.0006	Incremental cost per additional adequately diagnosed by CareStart compared to exclusive-use microscopy was USD 35550				
				RDT (ICT BinaxNOW) vs exclusive-use microscopy: USD - 20.26	RDT (ICT BinaxNOW) vs exclusive-use microscopy: - 0.0369	Incremental cost per additional adequately diagnosed by ICT BinaxNOW compared to exclusive-use microscopy was USD 549.05				
				RDT (First Response malaria combo) vs shared-use microscopy: USD - 0.55	RDT (First Response malaria combo) vs shared-use microscopy: - 0.0685	Incremental cost per additional adequately diagnosed by First Response Malaria Combo compared to shared-use microscopy was USD 8.03				
				RDT (Parascreen) vs shared-use microscopy: USD - 0.45	RDT (Parascreen) vs shared-use microscopy: - 0.1141	Incremental cost per additional adequately diagnosed by Parascreen compared to shared-use microscopy was USD 3.94				
				RDT (SD Bioline FK60) vs shared-use microscopy: USD - 0.44	RDT (SD Bioline FK60) vs shared-use microscopy: - 0.0767	Incremental cost per additional adequately diagnosed by SD Bioline FK60 compared to shared-use microscopy was USD 5.74				
				RDT (CareStart) vs shared-use microscopy: USD 2.49	RDT (CareStart) vs shared-use microscopy: - 0.0006	Incremental cost per additional adequately diagnosed by CareStart compared to shared-use microscopy was USD - 4150				
				RDT (ICT BinaxNOW) vs shared-use microscopy: USD 3.56	RDT (ICT BinaxNOW) vs shared-use microscopy: - 0.0369	Incremental cost per additional adequately diagnosed by ICT BinaxNOW compared to shared-use microscopy was USD - 96.48				
Osei-Kwakye 2013 [38]	Patient	12 months	The number of cases correctly diagnosed	RDT vs presumptive: USD 1.00	NR	The incremental analysis was not performed.	Sensitivity analysis for the incremental analysis was not performed.		NR	NR
				RDT vs microscopy: USD - 1.50	NR					
Shillcutt 2008 [39]	Provider and patient	NR	DALYs averted	NR	NR	NR	RDT was constantly more cost-effective compared to microscopy and the cost-effectiveness of RDT compared to presumptive diagnosis was sensitive to malaria prevalence, ACT cost, adherence to antibiotics, whether the illness was bacterial, whether a patient diagnosed as not having malaria received antibiotics and policy-maker’s willingness to pay.	USD 150	USD 2002	NR
Tawiah 2016 [40]	Societal	2 years	The number of appropriately treated children	RDT vs presumptive: USD 1474 per 1000 fever episodes	RDT vs presumptive: 134 per 1000 fever episodes	Incremental cost of introducing RDT to replace presumptive diagnosis per additional appropriately treated child under five from a societal perspective was USD 11.0	Univariate sensitivity analysis: ICERs from health sector and societal perspective were sensitive to adherence to RDT results, malaria prevalence, RDT specificity. Bivariate sensitivity analysis: ICER was insensitive to the accuracy of RDT. Probabilistic sensitivity analysis: Always improved effects but an increase in costs from a health sector perspective and uncertainty in costs from a societal perspective.	NR	USD 2011	5%
	Health sector			RDT vs presumptive: USD 2492 per 1000 fever episodes		Incremental cost of introducing RDT to replace presumptive diagnosis per additional appropriately treated child under five from a health sector perspective was USD 18.6.				
Uzochukwu 2009 [41]	Provider and patient	NR	Deaths averted based on the use of the alternative diagnostic strategies	RDT vs presumptive: USD -27 960 per 100 000 malaria cases	RDT vs presumptive: 127 per 100 000 malaria cases	Incremental cost of introducing RDT to replace presumptive diagnosis per death averted was USD - 221.	ICER was sensitive to malaria prevalence, the proportion of non-malaria febrile episodes that were bacterial, sensitivity of RDT, adherence to ACT, the cost of RCT and the cost of ACT.	NR	USD 2008	NR
				RDT vs microscopy: USD -56 781 per 100 000 malaria cases	RDT vs microscopy: 11 per 100 000 malaria cases	Incremental cost of introducing RDT to replace microscopy per death averted was USD - 5162.				

*NR* Not report, *SNMCP* The Senegalese National Malaria Control Programme, *AL* Artemether-lumefantrine, *ACT* Artemisinin-based combination therapy, *RDT* Rapid diagnostic test, *ICER* Incremental cost-effectiveness ratio; *DALY* Disability-adjusted life year

### RDT vs microscopy

Microscopy is a conventional diagnostic method to detect malaria infection. Six out of fifteen studies found that introducing RDT to substitute microscopy was likely to be cost-effective [27, 29, 35, 37, 39, 41]. Four of them made that conclusion as RDT could lead to either lower costs and improved outcomes, or a cost-saving when compared to microscopy [29, 35, 37, 41]. A cost-effectiveness analysis based on decision tree compared RDT and microscopy to presumptive diagnosis simultaneously [27]. It found that overall, RDT had lower positive ICER than microscopy and was most cost-effective in both high and low transmission settings. A decision-analytical study presented evidence of the cost-effectiveness of RDT compared to both microscopy and presumptive diagnosis [39]. With a threshold of USD 150 for the incremental cost per addition averted disability-adjusted life years (DALYs), RDT was highly likely to be cost-effective.

### RDT vs presumptive diagnosis

The cost-effectiveness of RDT in comparison to the presumptive diagnostic method was reported in ten studies, and all of them used presumptive diagnosis as a base case with RDT as the intervention to compare [27, 29–32, 34, 38–41]. Eight studies provided supportive evidence that RDT was highly likely to be cost-effective: three studies observed that the use of RDT could be less costly while more effective [27, 32, 41], three studies found that RDT could result in an increase in both costs and effectiveness but it had the potential to be cost-effective at a low willingness to pay (WTP) threshold [29, 30, 40], another study observed a low ICER of RDT but admitted that whether RDT could be cost-effective would depend on how much decision-makers would be willing to pay [31], and a decision-based analysis showed that RDT was 85% certain to be cost-effective at all prevalence level below 65% [39].

### Perspectives

Studies took a wide range of study perspectives which determined the scope of costs and effects within the evaluations: five studies were conducted from the societal perspective, four adopted a perspective of the health sector, one study did not report its perspective and the rest were undertaken under narrower perspectives such as provider or patient. There was a high level of heterogeneity among the selection of outcome measures among studies with narrow perspectives while the five studies under a societal perspective adopted either the number or the proportion of appropriately treated patients as the outcome, which can be considered as the same measure of effectiveness. We thus would take “the number of appropriately treated per 1000 suspected cases” as the main outcome and recalculate the results based on the available data.

**Fig. 3:**
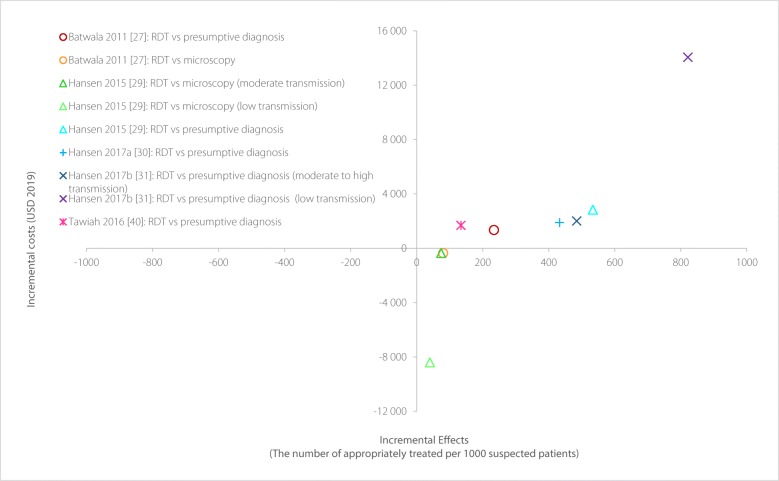
Incremental cost-effectiveness ratio of studies included (societal perspective) Each point represents differences in the costs and effectiveness between RDT and its alternatives from included studies under a societal perspective. RDT: Rapid diagnostic test.

The comparison of the economic value of RDT between five studies taking a societal perspective was plotted in Fig. 3. Compared with other diagnostic techniques, the incremental effects of RDT were always positive, i.e., using RDT could contribute to an increase in the number of appropriately treated patients, but its impact on additional societal costs was not clear and could largely depend on the comparator selected. The introduction of RDT to replace presumptive diagnosis resulted in an increase in costs [27, 29–31, 40], but that increase was relatively small in most of the studies. There were two studies that provided evidence for the comparison between RDT and microscopy from a societal perspective, they observed a cost-saving effect when RDT was introduced [27, 29]. Overall, given a small number of studies, it could be found that RDT had the potential to be cost-effective particularly compared to microscopy under a societal perspective and whether RDT could be a dominant strategy would largely depend on the threshold of policymakers.

Similar trends could be observed when economic evaluations were undertaken under a health sector perspective. Most of them concluded that RDT was cost-effective when it was compared to microscopy and likely to be cost-effective when compared with the presumptive diagnosis. RDT can keep its advantages over microscopy with lower costs and more patients appropriately treated [29, 31, 40]. It can also largely improve the clinical performance but resulted in a slight increase in the health sector costs if it was used to replace presumptive diagnosis [29–31, 40]. Further details of cost-effectiveness in studies included could be seen in Additional files 2 and 3.

### Prevalence

Changes to the malaria prevalence tended to have an impact on the costs and effects of diagnostic methods. Thirteen studies recognized its potential influence on the cost-effectiveness of RDT compared to other methods but only eight of them formally investigated the uncertainty brought by malaria prevalence [27, 30, 31, 33, 37, 39–41]. The introduction of RDT to replace microscopy was found to be a dominant strategy regardless of the prevalence levels [27, 37, 39, 41], but the ICER could be lower with an increase in prevalence [33]. The cost-effectiveness of RDT against presumptive diagnosis was consistent: all the four studies that tested the robustness of the results found that RDT could be more cost-effective in the area with lower prevalence [30, 31, 39, 40].

### Age

Among all included studies, eleven had no restriction on participants’ age and four limits the population to students or children of different ages. Evidence showed that whether RDT could be cost-effective compared to other diagnostic methods was not likely to be influenced by the age of the target population. Of the four papers with a limitation on the age, half applied RDT on children under 5 years old and supported the cost-effectiveness of this diagnostic method [31, 40], while the other half focused on children as well and did not reach that conclusion, but both of them recognized the cost-saving effect of RDT compared to microscopy [28, 38]. Of eleven studies without a limitation on age, eight showed that RDT could be more cost-effective compared with other methods [27, 29, 30, 32, 35, 37, 39, 41]. The majority of economic evaluations included considered RDT as a cost-effective strategy regardless of whether the study limited the subjects’ age. Further details can be seen in Additional files 2 and 3.

### The types of RDT

There are various types of RDT: some of them can detect single *Plasmodium* species, some can detect multiple species and some can distinguish between different species [42]. The difference in the types may bring extra costs to the economic value of RDT as they may have different prices. To compare the impact of RDT types, we categorized RDT into two categories: one is a single test which only detects single species, another is a combo test which can detect multiple *Plasmodium* species. The types of RDT used in included studies varied greatly. Ten studies adopted single test [27, 28, 30–35, 39, 40], while combo tests were used in seven studies [28, 29, 32, 36–38, 41].

Evidence suggested that single RDT could be cost-effective compared to microscopy and presumptive diagnosis. *Plasmodium falciparum*-specific RDTs were adopted in eight studies: four of them were decision analytical economic evaluations and suggested that the introduction of single RDT tests can largely improve the proportion of appropriate treatment for patients [27, 30, 31, 40]. In the other four studies, two of them found that RDT was likely to be more cost-effective than microscopy [35, 39], and the remaining two studies adopted single and multiple tests at the same time. In the first study conducted in Ethiopia where *P. falciparum* and *P. vivax* co-exist, both single and multiple tests were used to appraise the cost-effectiveness of RDT compared to presumptive treatment [32]. In the area with various malaria species, multiple tests were more cost-effective than either a single test or presumptive diagnosis. In a second study, a cost analysis was performed to appraise the performance of four RDT brands, including single and multiple tests, but it did not assess the effectiveness of multiple tests and only reported costs of general RDTs rather than costs by each RDT type [28].

However, the cost-effectiveness of combo tests was not clear. Four of seven studies showed positive results regarding the cost-effectiveness of combo RDT. Three studies that appraised the costs and effectiveness of RDT based on decision models observed lower costs and more clinical benefits with the use of multiple tests than microscopy [29, 37, 41]. Lemma et al. found that multiple tests performed better and cost lower than both single tests and presumptive diagnoses in the context where *P. falciparum* and *P. vivax* co-dominate [32]. However, the cost-effectiveness of multiple tests applied in the remote area of Amazon where *P. falciparum* and *P. vivax* dominate as well were uncertain as it largely depended on the accessibility to and the accuracy of microscopy [36]. Evidence identified in this review observed that RDT could also lead to the problem of over-diagnosis [28, 38]. Although RDT was the cheapest approach to detect infection in malaria school surveys compared to other strategies (i.e., microscopy or RDT corrected by alternative methods), it over-estimated the prevalence of infection [28]. Also, the study only evaluated the costs of diagnosis and thus the cost-saving effect of RDT could be maintained remainsed unclear when treatment costs were taken into account. The treatment costs were found to be higher for RDT than for microscopy when *P. falciparum* and *pan*-specific RDT was used to the management of malaria cases in Ghana [38]. The study also observed the over-diagnosis and additional costs when RDT was introduced to replace presumptive diagnosis. This may reduce RDT’s advantage in terms of cost-effectiveness.

In general, the impact of the types of RDT on its cost-effectiveness remained uncertain given various types of RDT, the complexity of local epidemiological characteristics and the lack of evidence reported in studies included. Further details of the types and brands of RDT can be seen in Table 1.

### Funding sources

Fourteen of all fifteen studies received funding from various sources (Additional file 4). It was not clear based on current evidence whether founding sources would have an impact on whether RDT was cost-effective. Seven studies were government-sponsored, either intergovernmental organization or local government [28, 32, 34, 35, 37, 39, 41], and five of them supported the cost-effectiveness of RDT [32, 35, 37, 39, 41]. Of eight studies that did not receive funding from the government [27, 29–31, 33, 36, 38, 40], seven were sponsored by either non-governmental organizations or research institutions including universities and five studies reported that RDT was cost-effective [27, 29–31, 40]. There was only one study that had no statement of the source of funding, and its result did not support RDT’s cost-effectiveness because it found that if the accuracy of microscopy could be guaranteed, there would be no additional benefits of applying RDT [36]. As most of the studies included received funding from nonprofit organizations and there was only one research that did not report its funding source, the impact of funding sources was less clear.

## Discussion

### Economic evidence for RDT

Our study aimed at assessing the cost-effectiveness of RDT in a systematic manner. Overall, we identified fifteen studies that tried to delve out whether RDT was cost-effective compared with other commonly used malaria diagnosis methods and there was heterogeneity in population age, funding sources, economic and effectiveness measures, and other general study settings across studies. Our analysis took the influence of such variability into account and found that most studies provided supportive evidence in terms of the cost-effectiveness of RDT.

However, there were still five studies that did not draw a clear conclusion [28, 33, 34, 36, 38]. This difference can be explained by the accuracy of RDT, the performance of its comparisons, clinicians’ compliance with the diagnostic results, total treatment costs, and malaria prevalence. Therefore, we were unable to conclude which strategy would be the most cost-effective with certainty.

A wide range of perspectives has been selected by the studies included. Although most of the studies under the societal and health sector perspectives supported the cost-effectiveness of RDT, some could not because of the uncertainty in the costs and an unclear WTP threshold. This might suggest that the diagnosis and treatment of malaria can be unaffordable to patients in many countries. The recommended first-line malaria treatment, artemisinin-based combination therapy (ACT), is expensive and possible increase in treatment costs over time due to therapy resistance and drug prices has been seldom considered by researchers in the field trials. A cost-effective intervention can be considered to receive public funding if it is a public good, or has important externalities and inadequate demand, or is catastrophically unaffordable and has no available insurance, or beneficiaries are poor when utility outcome is not available [43]. Given the expensive treatment costs, it is suggested that malaria case management with RDT should be included in the coverage of health insurance to substantially reduce the economic burden on patients and their families [44, 45].

Another key driver for the cost-effectiveness of RDT is its price [27, 30, 31, 33, 36, 41]. The price of RDT can be determined by its type as combo RDTs are usually more costly than the single tests. In our analysis, it is uncertain whether the cost-effectiveness of RDT could be influenced by the types of RDT. Also, the capability of combo tests to identify plasmodium species can largely influence the cost-effectiveness of RDT because the type of RDT selected will determine not only the accuracy of diagnosis [46], but also the following treatment received. Therefore, for most countries where multiple malaria species dominate, it is necessary to differentiate Plasmodium species such that proper treatment could be delivered.

This systematic review included studies from low- and middle-income countries that were assumed to be malaria endemic. The cost-effectiveness of RDT compared to microscopy was not clear in regions with relatively low transmission settings given the uncertainty in how the routine microscopy was performed, i.e., the accuracy of microscopy and whether the microscope was used only for malaria detection. Current evidence suggested that RDT could be more cost-effective than microscopy [29, 37, 39, 41], and the relative advantage of RDT could be further enhanced if microscope was exclusively-used [37]. This could be explained by the fact that the demand for malaria diagnosis would be less in area where malaria prevalence is close to zero, and the cost per suspected patient would be largely increased when taking microscopy as the initial approach. Further studies are required to confirm this, especially in low transmission countries aiming at eliminating malaria.

Moreover, facing the reduction of malaria prevalence and movements towards disease elimination [47], it is more common for countries to confront the threat of increasing malaria imported cases [6]. Usually, imported patients are either rural migrant workers or travelers to the endemic region, and tend to have lower parasite densities. The key challenge is to promptly and accurately identify malaria cases at all levels of health systems. Current malaria control programs have established either active or passive case detection systems. Active case detection requires health workers to seek out for patients, making it less feasible to maintain the use of microscopy as the initial approach when the prevalence is extremely low. In addition to this, our requirements for malaria elimination, especially a consistent diagnostic accuracy for community-based primary care, is beyond the capacity of routine microscopy due to the scarcity of well-trained microscopists [48], and essential laboratory supplies. This may limit the performance of microscopy [49], and contribute to misdiagnosis or over-diagnosis with a potential risk of over-consuming antimalarial therapies and drug resistance [50]. In fact, the poor performance of routine microscopy has been widely recognized, even in developed countries [51], and high capital investment of microscopy makes it more costly than RDT if local caseload is low [52]. Therefore, it is meaningful and economically important to introduce RDT in primary health care or remote region where microscopy is unavailable.

### Quality of the evidence

The CHEERS tool was used to assess the quality of evidence in our research, allowing to compare reporting quality across included studies. The majority of studies identified are of good and moderate quality, but we still found some studies showed poor compliance with the reporting guidance, especially lacking details of research methods. Explanation of model selection was lacked generally, and this might be because studies tended to have more concern about whether RDT was a quick and accurate way to detect malaria cases. It should also be noticed that the scores of studies aimed at measuring the cost-effectiveness of RDT were higher than those only evaluating the costs of the disease detection approaches.

In addition, the CHEERS tool focuses on the quality of reporting, and it should be fully considered at the stage of study design, for example, by referring to the structural abstract proposed by NHS Economic Evaluation Database (NHS EED) and extracting basic characteristics and results of health economic evaluations to improve the quality of evidence.

### Limitations

This review is limited in the following aspects: firstly, the studies identified were conducted in a limited range of countries, most of which were located in Africa. Whether results obtained from the context can be transferable to other countries was not elaborated in the included studies. It is obvious that countries may vary in their widely-used malaria diagnostic methods and other features such as prevalence and the types of RDT. Differences in health care systems and reimbursement also limit the transferability of our results. Thus, caution should be taken when applying the results to other settings. Another limitation was inadequate data on costs and effectiveness, possibly due to differences in adopting primary and secondary outcome indicators among studies, adding to the difficulty in comparing ICERs obtained when they had the same perspectives. Therefore, no synthesized outcome was shown due to the wide difference across studies and the lack of evidence regarding health utility gained when using different malaria diagnostic techniques. We suggest that further economic evaluations of malaria detection methods should focus on health utility benefits for patients who are susceptible to the disease.

## Conclusions

We compared the cost-effectiveness of malaria RDT to other conventional diagnostic methods based on fifteen economic evaluations identified. However, there was high heterogeneity across economic evaluations identified in the outcome measures selection, the use of comparative diagnostic methods, and study settings. RDT was highly likely to be consistently cost-effective compared to presumptive diagnosis and routine microscopy, particularly in a low transmission setting. Further economic evaluations with better quality and comparable study designs were required.

## Supplementary information

**Additional file 1.** Methodological quality of all included studies.

**Additional file 2.** Economic results of the included studies: effectiveness measures, prevalence, original ICERs and adjusted ICERs (USD 2019).

**Additional file 3.** Economic results of the included studies: interventions, economic results and conclusions.

**Additional file 4.** Perspectives, funding sources and conflicts of interests of studies.

## Data Availability

All data generated or analyzed in this study are included in this published article and its supplementary information files.

## Abbreviations

ACT: Artemisinin-based combination therapy
CHEERS: Consolidated Health Economic Evaluation Reporting Standards
PCR: Polymerase chain reaction
RDT: Rapid diagnostic test
WHO: World Health Organization
